# Supplementary material for: Full-length genome characterization and phylogenetic analysis of SARS-CoV-2 virus strains from Yogyakarta and Central Java, Indonesia
Source: PeerJ. 2020 Dec 21;8:e10575. doi: 10.7717/peerj.10575 (PMC7759132; doi:10.7717/peerj.10575)
Supplement: Supplemental Information 2 [file peerj-08-10575-s002.docx]

**Supplementary Table 1.** Presentation of COVID-19 cases from Yogyakarta and Central Java.

***Case 1***

An 83-year-old female patient complained of fever 13 days before admission. She had a history of contact with a COVID-19 confirmed case, and RT-PCR tests were conducted on August 10, 2020, with positive results. She had comorbidities of hypertension, geriatric syndrome, and congestive heart failure. The physical examination recorded a blood pressure of 150/90 mmHg, with normal results on her remaining vital signs. Chest X-rays showed the appearance of infiltrate on both lungs. She was diagnosed with moderate COVID-19 and mild pneumonia. After admission, the patient received antibiotics and antiviral therapy based on the COVID-19 Prevention and Control guidelines by the Indonesian Ministry of Health, namely, azithromycin and oseltamivir. The patient was uneventfully discharged from the hospital 29 days after admission.

***Case 2***

A 77-year-old male patient complained of dry cough. He had a history of contact with a COVID-19 confirmed case two weeks before admission. RT-PCR tests were conducted on June 22, 2020, with positive results. He had a comorbidity of gout arthritis. The physical examination recorded a blood pressure of 120/80 mmHg, pulse of 68 per minute, respiratory rate of 20 per minute, body temperature of 36.6°C, and oxygen saturation of 95% with room air. Lung auscultation revealed crackles posteroinferior to the lung. Chest X-rays showed no abnormality, but thoracic CT scan revealed infiltrate and ground glass opacities on the bilateral posteroinferior lung, typical of viral pneumonia caused by COVID-19 infection. We found increases in the neutrophil-to-lymphocyte-ratio and uric acid of 3.11 and 8.9 mg/L, respectively. He was diagnosed with moderate COVID-19 and mild pneumonia. The patient received azithromycin and hydroxychloroquine. He was uneventfully discharged from the hospital 20 days after admission.

***Case 3***

A 55-year-old female presented with complaints of cough that were experienced from one week before admission. Positive RT-PCR results were obtained on June 26, 2020. The patient had comorbidities of diabetes mellitus. Her vital signs are within normal limits. Lung auscultation revealed crackles in both lungs. Chest X-rays showed bilateral infiltrate. We found increases in blood glucose levels of 340.56 mg/dL. A blood culture test was performed and showed negative bacterial growth. She was diagnosed with moderate COVID-19 and mild pneumonia. The patient received antibiotics and antiviral therapy concordant with the COVID-19 Prevention and Control guidelines by the Indonesian Ministry of Health, namely, azithromycin, hydroxychloroquine, and oseltamivir. She uneventfully recovered and was discharged from the hospital 31 days after admission.

***Case 4***

A 30-year-old male came to the emergency department with a chief complaint of cough. He experienced sore throat and coughing up mucoid phlegm. The RT-PCR tests on SARS-CoV-2 upon admission were positive (conducted on May 16, 2020). His vital signs are within the normal range. Pulmonary auscultation was unremarkable. Chest X-rays showed no abnormality, while routine blood tests revealed lymphopenia. He had a history of traveling from the local COVID-19 transmission area. He was diagnosed with mild COVID-19. The patient received guideline-based therapy, namely, hydroxychloroquine and oseltamivir. The patient was discharged from the hospital 30 days after admission.
